# Visualization of Alternative Functional Configurations of Influenza Virus Hemagglutinin Facilitates Rapid Selection of Complementing Vaccines in Emergency Situations

**DOI:** 10.3390/ijms18040766

**Published:** 2017-04-04

**Authors:** Ashraf Metwally, Ausama Yousif

**Affiliations:** 1 Veterinary Virologist and Consultant, Cairo 11441, Egypt; drashrafmali@yahoo.com; 2 Virology Department, Faculty of Veterinary Medicine, Cairo University, Giza 12211, Egypt

**Keywords:** 3D modeling, alternative functional configurations, anchor sites, avian influenza virus, complementing structural units, escape mutants, fitness condition, hemagglutinin, vaccine combinations, vulnerable sites

## Abstract

Successful immunization against avian influenza virus (AIV) requires eliciting an adequate polyclonal response to AIV hemagglutinin (HA) subunit 1 (HA1) epitopes. Outbreaks of highly-pathogenic (HP) AIV subtype H5N1 can occur in vaccinated flocks in many endemic areas. Protection against emerging AIV is partly hindered by the limitations of vaccine production and transport, the use of leaky vaccines, and the use of multiple, and often antigenically-diverse, vaccines. It was hypothesized that the majority of alternative functional configurations (AFC) within the AIV HA1 can be represented by the pool of vaccine seed viruses currently in production because only a finite number of AFC are possible within each substructure of the molecule. Therefore, combinations of commercial vaccines containing complementing structural units (CSU) to each HA1 substructure can elicit responses to the totality of a given emerging AIV HA1 substructure isoforms. Analysis of homology-based 3D models of vaccine seed and emerging viruses facilitated the definition of HA1 AFC isoforms. CSU-based plots were used to predict which commercial vaccine combinations could have been used to cover nine selected AFC isoforms on recent Egyptian HP AIV H5N1 outbreak viruses. It is projected that expansion of the vaccine HA1 3D model database will improve international emergency responses to AIV.

## 1. Introduction

The introduction of highly pathogenic (HP) avian influenza virus (AIV) H5N1 into new geographical locations has triggered intensive control efforts to curb its severe economic impact [1], and threat to public health. However, it is often difficult to contain AIV outbreaks for reasons related to (1) the nature of the virus; (2) its epidemiological characteristics; and (3) vaccine design and application. Failure to eradicate the virus immediately after its introduction results in establishment of an endemic state. Continued virus replication in endemic areas, and the spread from infected birds to other birds and other species in infected zones, increases the risk of generating potentially pandemic AIV [2]. 

AIV is an influenza A virus (IAV) of *Orthomyxoviridae*. It has a segmented negative-sense single-stranded RNA genome. The translation products of segments 1–3 assemble into the viral transcriptase complex [3]. The rapid evolutionary pattern of AIV is a result of its high rate of transcriptase errors, recombination, and reassortment [3,4]. This rapid mutation is sometimes associated with adaptation to multiple host species (including humans, animals, and birds) [3]. It can also lead to change in virulence characteristics. The existence of susceptible wild animal and bird species adds further layers of complexity to AIV epidemiology [5].

It can be argued that AIV exists as a quasispecies pool due to the antigenic and genetic diversity of progeny virions generated during infection [6]. Selection of particular phenotype(s) of the quasispecies pool is driven by population immunity and adaptation for replication in a particular host [6]. Mutants associated with particular environments/geographical locations have been observed [7,8].

AIV H5 vaccines are usually used to protect chickens, reduce virus load in the environment, and prevent transmission of H5N1 viruses from poultry to humans [9,10]. AIV H5 vaccines in production today include inactivated AIV generated using reverse genetics approaches, and recombinant viral vector vaccines [11]. The viral hemagglutinin (HA) is the main immunogen in AIV vaccines [3,12].

IAV HA precursor (HA0) is a 76 KDa glycoprotein that is encoded on segment 4 of the AIV genome. HA is present as trimers on the envelope [3]. Eighteen influenza A virus HA subtypes exist [11]. During infection, influenza virus HA0 is cleaved by cellular proteases into two subunits, HA1 (47 KDa) and HA2 (29 KDa). The HA is responsible for virus attachment to host cell receptors, and for penetration by fusion after conformational changes in the endolysosomal compartment [3,13].

The multifaceted function of the HA timers is facilitated by conservation of the HA molecule shape, adaptability of the binding sites, and flexibility of segments involved in fusion events. These characters are largely dependent on electrostatic interactions at interfaces, such as hydrogen bonds and salt bridges, and van der Waal interactions. These interactions are important for keeping the metastable conformation at neutral pH [13].

Surface structures of the IAV HA are primary targets of neutralizing antibodies (nAbs) [14], and are involved in the phenomenon of antigenic drift [15]. One or more subpopulations of nAbs directed to specific conformational epitopes on the globular head of viral HA1 fail to bind when escape mutants are generated [16,17]. The latter epitopes have been recently allocated to four vulnerable sites (VS1-4) on the globular head using structure-function analysis utilizing sera of convalescent patients and genetic polymorphism studies. Vulnerable sites on the AIV H5N1 correspond to those identified earlier on the HA of H1 and H3 subtypes. The total surface area for VS1-4 constitutes up to 52% of the globular head HA ectodomain. Mutations within the vulnerable sites were associated with escape mutant generation [17].

AIV vaccine seed viruses are selected to resemble the majority population of circulating viruses [11]. Antigenic variation among circulating HP AIV H5N1 mandates the continuous production of strain-specific pre-pandemic vaccine candidates [18]. In some endemic regions, the logistic situation prevents the exclusion of influenza vaccines that contain an H5 antigenic component that is not highly homologous to the H5 of local field strain(s); thus, multiple, and often antigenically-diverse vaccines are used [5,11,19]. Since protection conferred by such vaccines is incomplete, efficient virus replication and shedding continues and results in the virus leaking into the environment; hence, the term “leaky vaccines” [20].

Incorrect application of vaccine-based control strategies contributes to AIV diversity and results in complication of the epidemiological situation. The Egyptian situation is a clear example of this problem. Although vaccination against AIV H5N1 was adopted in Egypt since 2006, severe avian influenza (AI) associated with HP AIV H5N1 continue to be reported in vaccinated flocks [5,21]. Phylogenetically-divergent H5N1 viruses, which are only partly antigenically cross-reactive, co-circulate in Egypt [22,23]. The Egyptian situation poses a threat to the rest of the world [5].

In endemic developing regions, the current practices of vaccine seed update, production, transport to outbreak areas, and application will continue to favor emerging virus escape and evolution. In most developing countries, responses to emerging viruses are often too late and too little to effectively restrict the spread of emerging mutants [5,11].

Understanding two biological foundations of virus infectivity may offer solutions applicable to control of emerging viruses in endemic situations (i.e., within a given endemic virus subtype). The first of these foundations is that structure-function relationships of the HA must be conserved (condition 1). If independent evolution of the different segments of the viral HA molecule occurs in a manner that may affect function, then virus survival can only be achieved if compensatory mutations occur to restore function (fitness) [6]. Second, escape from population immunity is simpler (more efficient) by selecting mutants that are not recognized by existing immunologic memory [6,24] (condition 2). Therefore, rather than accumulating a large amount of mutations to evolve novel functional structures, it is easier to just switch between a repertoire of acceptable substructures that maintain the function. This means that HA antigenic structures that fulfill conditions 1 and 2 may not be necessarily novel, but rather novel for the virus under the existing immunological or environmental pressure.

Examples demonstrating the correctness of conditions 1 and 2 include the conservation of structural/functional units found within the H5N1 HA receptor binging site (RBS) and fusion peptide. Translation of this concept means the possible conservation of other biologically important substructures of the HA1 molecule within the quasi-species population. If these biologically-important substructures can be defined, then it would be possible to assemble the appropriate substructure pool from current vaccines to produce the polyclonal response necessary to protect against emerging viruses. In other words, it may be possible to define the foundation of using vaccine cocktails.

It was the objective of this investigation to test if the AIV HA1 biologically/immunologically-important substructures can be defined using current bioinformatics approaches, and whether the technology can be used to devise a possible new vaccination approach for use against emerging mutants in endemic areas.

## 2. Results and Discussion

### 2.1. Hypothesis Developoment

The emergence of divergent Egyptian H5N1 AIV mutants has been observed as early as 2007 [25], despite the implementation of massive vaccination [26]. Protection against emerging/reemerging AIV mutants in endemic areas is partly hindered by one or more of the following factors: (1) time required to update vaccine seed; (2) vaccine production capacity; (3) the time required to import vaccines from international suppliers; (4) the use of “leaky vaccines” [20,27]; and (5) the use of multiple, and often antigenically-diverse, vaccines [5,7,19]. The use of leaky vaccines and multiple antigenically-diverse vaccines allows for virus replication and subsequent mutation in partially-protected flocks. This situation can be exacerbated with improper and/or uneven application of vaccines in endemic regions, and poor flock conditions (e.g., poor feed quality, housing, management, health status, etc.) [11,26]. On the other hand, rethinking the way this plethora of commercial vaccines is used in endemic areas may provide an opportunity to limit the impact of virus diversity, particularly in emergency situations.

Vaccine antigens are viewed as epitopes by the host’s immune system. These epitopes may be linear or conformational. Successful clearance of virus infections is linked to the ability of the immune system to recognize and mount an effective response to critical neutralizing and cytotoxic epitopes. Vaccine formulations are designed to enhance the development of protective responses at the level and location required to successfully break the cycle of infection in hosts and populations [9,10,27]. Vaccines are only licensed for commercial use if they pass rigorous efficacy testing by producers and regulatory bodies [11]. Therefore, it is only logical that each of these licensed vaccines contains the pool of epitopes that effectively represents the challenge virus; otherwise, it would not have passed efficacy testing.

AIV mutants that escape the immune response generated by vaccines need only change certain structural units (epitopes) within the HA1 globular head [17]. In effect, the vaccine shield drives the disequilibrium within the quasi-species spectrum [6]. However, mutations will be selected against if they alter structural units of the virus required for survival within the environment [6]. The preservation of the AIV HA molecule shape and function is a good example of this “fitness condition” (FC). A significant increase was observed in the amino acid substitution rate of the HP AIV H5N1 HA1 subunit, but not the HA2 subunit, during periods of mass vaccination [16,17]. This means that multiple alternative functional configurations (AFC) are possible (comply with the FC) within this region of the AIV HA. Nevertheless, this pool of AFC must be finite to preserve the FC. A change outside this finite number of AFC would either lead to loss of fitness, or the generation of a new subtype of HA. The latter probability is not the subject of this discussion.

It is hypothesized that the majority, if not all, of the HA1 AFC can be found in the pool of vaccine seed viruses that has been subject to update for decades. Moreover, due to the worldwide HP AIV problem, the AFC may already be represented by the pool of vaccine seed viruses currently in production. If indeed this was the case, then an alternative strategy for current vaccine use can be devised to reduce the impact of evolution and co-circulation of AIVs.

### 2.2. One-Dimensional Analysis Is Not Appropriate for Alternative Functional Configurations (AFC) Recognition

**Figure 1:**
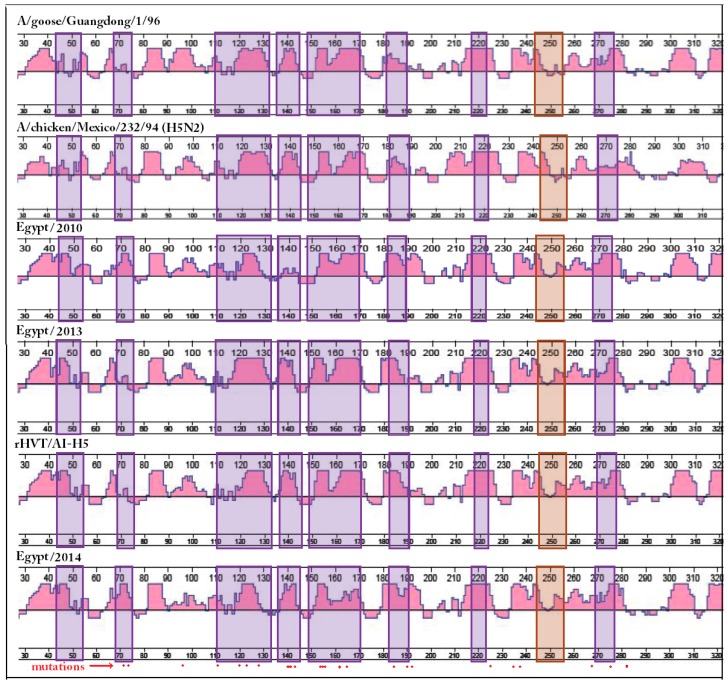
The antigenic index of avian influenza virus hemagglutinin subunit 1 (AIV HA1) protein sequences. The antigenic index was calculated and plotted using Protean™ (DNA Star, Lasergene^®^, version 7.1.0, DNAStar Inc., Madison, WI, USA). The antigenic index was used because of its relevance to vaccine design and evaluation. Sequence elements involved in vulnerable sites (VS) formation [17] were highlighted using violet boxes. The linear epitope sequences [28] were highlighted using a light brown colored box. The H5 numbering system was used. Red dots below indicate amino acid differences between the vaccine seed and field viruses in Case C (Section 3.1). The antigenic index was affected with mutations inside VS in a manner that was not always related to the number of mutated amino acids. Amino acid changes outside VS (Figure S1) that were also associated with changes in hydrophilicity and/or charge had a dramatic effect on adjacent VS.

The study of the individual amino acid mutations that occur during AIV HA evolution provides insight into the possible consequences of these mutations (i.e., change in hydrophilicity or charge of specific regions within the molecule) (Table S1). The majority of amino acid changes observed in the viruses studied (Figure S1) were associated with regions that are known to be exposed on the surface of the HA molecule (amino acid spans 43–53, 68–75, 110–145, 182–190, and 269–277). These regions were also linked to the VS associated with nAbs binding [17]. Amino acid substitutions within known nAbs binding positions [17,28] were sometimes accompanied by changes in hydrophilicity and/or charge (amino acids 43, 45, 74, 120, 123, 124, 129, 138, 140, 141, 144, 151, 154, 156, 158, 162, 165, 169, 183, 184, 188, 252, 270, 272, 275, and 276) (Table S1, Figure S1). Amino acid changes outside VS that were also associated with changes in hydrophilicity and/or charge had a dramatic effect on adjacent VS with no mutations. An example of this can be observed as changes of the predicted antigenic index (2D analysis) associated with mutation T108I of the recombinant herpesvirus of turkey expressing the AIV H5 vaccine seed virus (rHVT/AI-H5) and Egypt/2013 when compared to Guangdong/1/1996 (Figure 1). Therefore, one-dimensional (1D) analysis of HA1 is not appropriate for AFC recognition.

### 2.3. Two-Dimensional Analysis Is Not Suitable for AFC Definition

Two-dimensional analysis facilitated the visualization of how 1D analysis is not suitable for finding AFC. However, can 2D analysis be used as a tool for defining the proposed AFC? A study of vaccination escape cases could provide information on which mutations lead to structural changes that result in vaccination escape.

Severe AI associated with HP AIV H5N1 in vaccinated flocks can be attributed to (1) the inappropriate application of vaccines; (2) the emergence of vaccine escape mutants from endemic AIV strains; or (3) the introduction of heterologous AIV. In the flocks studied, the inappropriate use of vaccines was excluded as homogenous high antibody titers were reported following vaccination (Section 3.1). A nucleotide-based phylogenetic analysis was constructed to investigate which of the latter two was responsible for the outbreaks in the studied flocks.

In two cases, A and B, the outbreak viruses belonged to subclades 2.2.1.1 and 2.2.1.2, respectively. Both flocks in cases A and B were vaccinated using an H5N2 vaccine seed virus (Section 3.1). It was necessary to exclude the H5N2 vaccine seed virus from the phylogenetic analysis to avoid the loss of branch discrimination (Figure S2). In Case C, the situation was different. Hungary/4999/2006, the rHVT/AI-H5 (Section 3.1), is more closely related to the outbreak virus detected (Figure S2), and shows the least amount of amino acid mutations compared to the vaccine used (Table S1, Figure S1). Amino acid sequence identity percentages for viruses detected in flocks A, B, and C with their corresponding vaccines (Section 3.1) were 80.7%, 83%, and 94.2%, respectively. The corresponding sequence divergence values for viruses detected in flocks A, B, and C were 22.3, 19.3, and 6.1, respectively. The higher degree of homology between the vaccine seed and the outbreak virus in Case C indicated that it may be used in 2D analysis for AFC determination. 

The antigenic index was chosen for 2D analysis because of its relevance to vaccine design [29]. The antigenic indices of the detected viruses and commercial vaccine seed viruses described in this study were aligned to enable visualization of regions with conserved and varying values (Figure 1). Regions with conserved antigenic index values were observed within VS and areas outside VS (Figure 1). The location of the 24 amino acid changes between the outbreak virus of Case C and the vaccine strain used for immunizing the flock in Case C (Table S1, Figure S1) were marked on the antigenic index graphs to allow visualization of how mutations affect the analysis values (Figure 1). Out of the 24 marked amino acids, 17 were observed in known VS of the globular head (Figure 1).

Interestingly, the number of amino acid mutations within any segment of the vulnerable site was not always associated with the magnitude of change observed in the antigenic index. One prominent example would be the region 149–170 in Case C; where the first three mutations were associated with a significantly lower effect on the predicted antigenic index compared to the latter two (Figure 1). An additional mutation (E268K) in the Case C outbreak virus may have resulted in the change of the antigenic index values of the downstream VS fragment (Figure 1).

Taking in consideration that mutations within the VS are associated with neutralization escape in in vitro experiments [17], any alteration of the final structure (substructure) recognizable by the complementarity determining regions (CDR) of antibodies, even in the absence of mutation, could possibly have neutralization-escape consequences. Neutralization-escape could also happen as a consequence of 3D changes that would restrict the access of whole antibody molecules to the binding site, i.e., steric hindrance [30]. Mutations within conformational epitopes, or near them, may also affect the affinity of binding of preexisting nAbs; thus, further contributing to the phenomenon of leaky vaccines [20]. This quantitative effect on the sensitivity of the VS to antibody binding due to minimal amino acid mutations was observed using antisera of H5N1 AIV convalescent individuals [17]. Linear epitopes, e.g., 244–256 [28], are less subject to this interaction complexity. Nevertheless, the fact that most VS are composed of conformational epitopes (Figure S1) limits the use of 2D analysis for AFC determination; 2D analysis predicts changes in, or around, the mutation site, but is unable to show relationships formed by interacting secondary protein structures that are remotely located on the HA molecule (Protean™, DNASTAR, [31]).

### 2.4. Three-Dimensional (3D) Homology-Based Modeling That Conserves the Backbone of the Template Used for Comparison Can Be Used to Visualize the Effect of Cis- and Trans- Mutations on HA1 Surface Topology Elements

Definition of the conformations involved in biological functions, protective immunogenesis, or neutralization escape, is classically conducted using neutralization/inhibition/protection assays that involve the use of specific mAbs, convalescent antisera [17,32], or antibody cocktails [33]. These technologies should preferably be accompanied by determination of the 3D structures of the studied molecules [34,35]. Such studies are complex, time-demanding, and require heavy investment in infrastructure; making them prohibitive to most researchers in developing countries where most AIV problems exist. Furthermore, the evolution mechanism of AIV far outpaces our current ability to study and respond to mutations in a timely manner, even in developed countries [7,11]. Therefore, it is necessary to use rapid techniques for predicting the consequences of mutations as soon as an emerging virus is detected. Such information may then be used in the design of timely response strategies. Delay in generating this kind of information may amount to the loss of the battle for containment of the emerging strains.

Integrative structure solution techniques, including computational modeling, which can combine experimental data from heterogeneous sources and are able to handle ambiguous or conflicting information, are rapidly evolving [34]. Several homology-based and ab initio options for computational modeling of 3D structures exist. Ab initio modeling involves structure prediction based on amino acid sequence information. Homology-based techniques rely on a known template(s) for modeling. The latter is increasingly being accepted by both the modeling and wider bioscience communities as the most reliable approach for modeling [36]. Current comparative modeling techniques are automated and easily accessible to non-specialists [34].

Several experimentally-determined 3D structures of the AIV H5 molecule have been reported (Protein Data Bank “PDB”) [37,38]. Therefore, it is expected that modeling based on this dataset can be highly reliable. However, current modeling solutions adopt different approaches to solving the question of how to combine information from multiple templates, e.g., different structural domains, into larger complex assemblies. Current techniques predict the relative orientation of domains of multi-template models with varying degrees of reliability [34]. Therefore, selection of the modeling software to use in 3D modeling had to rely initially on results of worldwide blind evaluations using difficult and easy targets (Critical Assessment of protein Structure Prediction “CASP”) [39]. 

I-TASSER [40] has consistently been shown superior in difficult molecule 3D structure resolution [36,41,42]. However, very few differences were observed between different modeling servers when easy molecules were used for comparison [36,41]. Modeling using I-TASSER [40] generated several probable 3D alternative models (Figure S3). These multiple alternative folding patterns and interactions with solvent were observed on both the HA1 and the stalk regions of the modeled molecules (Figure S3). Such alternatives are expected; the flexibility of certain regions within the molecule is essential for its function, e.g., allosteric conformational changes on binding events. This same finding supports the concept of AFC of flexible regions at the level of an individual virus [13,14,34].

**Figure 2:**
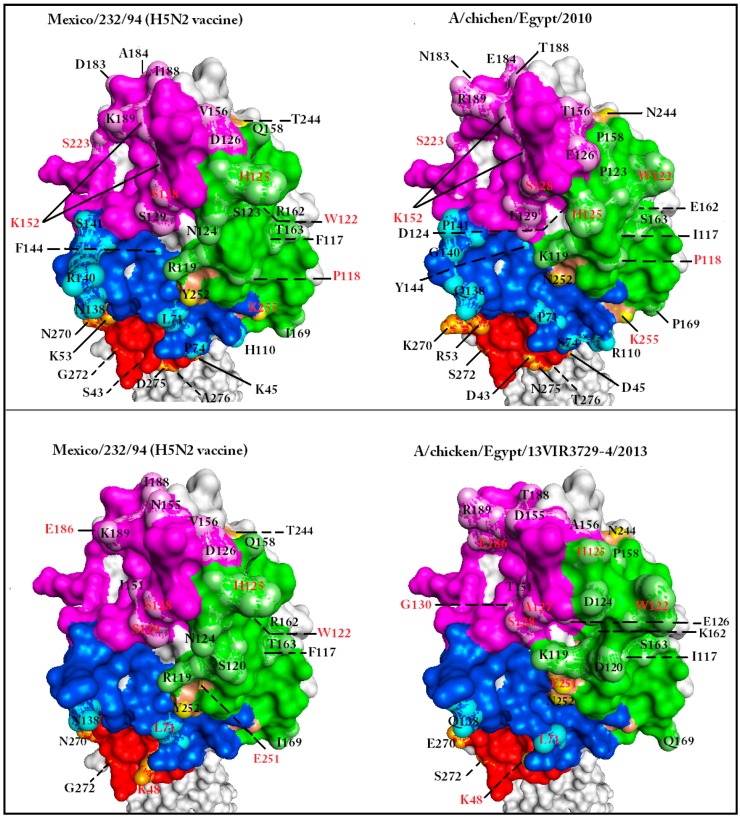

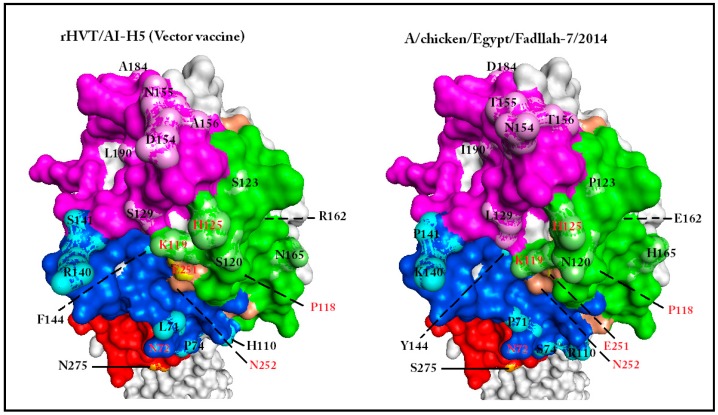
The effect of amino acid mutations in or around vulnerable sites on the AIV HA1 solvent surface topology. Three-dimensional models of whole hemagglutinin (HA) molecules were generated using Phyre2 (a web portal for protein homology-based modeling, prediction, and analysis) [36,45]. Modeled structures were visualized using Accelrys^®^ Discovery Studio v4.1 software (Dassault Systèmes BIOVIA Corp., San Diego, CA, USA) using solvent surface structure view. The different models were synchronized to unify orientation. Only the HA1 globular head and part of the stalk are shown. Sequence elements involved in VS formation were colored green, magenta, blue, and red according to the original coloring code of VS1-4, respectively [17]. The linear epitope sequences [28] were colored bronze. The locations of amino acid mutations and/or surface alterations were highlighted using a bright coloration of background of each region. When comparing each case, the mutated amino acids were indicated by solid lines on the 3D model. Amino acid identifications in red indicate positions showing surface alterations without amino acid mutations. Dashed lines indicate the position of amino acid mutations that are not exposed in the present view. The presented view of the 3D models illustrate that specific substructures of the molecule exhibit conserved confirmation with or without mutation. Other substructures show topology variations due to mutations in or around the substructure.

The I-TASSER [40] strength adds complexity to HA1 AFC determination. Therefore, we resorted to a counterintuitive point of strength characteristic of many homology-based servers to simplify AFC definition. Homology-based models conserve the backbone of the template used for comparison, and point mutations are presented only as changes in side-chain positions [36]. It was argued that this reported limitation, combined with a solvent accessibility view of the model, will show enough structural conservation to enable the definition of individual substructures exhibiting AFC. The solvent accessibility view shows the interface with the medium in which the function is performed [43,44]. This approach for substructure visualization does not necessarily mean that the visualized model is completely identical to the native molecule at a given point of time and space. Phyre2 [45] was one of the modeling software that generated models with the required characteristics [46], and was selected to generate the different models used for comparisons between the studied vaccine seed and field viruses (Figure 2).

A comparative study of six modeled HA molecules revealed several features. The FC was expressed in the form of conserved 3D substructures in all studied HA models (Figure 2). Additionally, some amino acid mutations (e.g., S123P in Case C) were not accompanied by distinct surface alterations. Moreover, conserved regions were generated as a result of structure compensation following deletion (S129 deletion of Egypt/2013) (Figure S1).

### 2.5. Alternative Functional Configurations (AFC) Can Be Visualized Using Anchored Homology-Based 3D Models in Solvent Accessibility View

**Figure 3:**
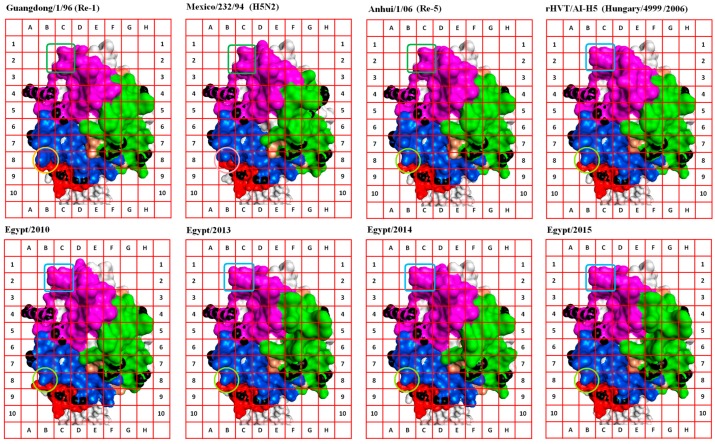
Visualization of AIV HA1 alternative functional configurations (AFC). Three-dimensional models of whole hemagglutinin (HA) molecules were generated using Phyre2 [45]. The whole HA sequence was used for modeling. The models were visualized using Accelrys^®^ Discovery Studio v4.1 software (Dassault Systèmes BIOVIA Corp., San Diego, CA, USA, Section 3.5). Sequence elements involved in VS formation were colored green, magenta, blue, and red according to the original coloring code of VS1-4, respectively [17]. The linear epitope sequences [28] were colored bronze. Models were synchronized and trimmed to present an optimum view of the VS. Conserved substructures identified as anchor sites (AS) (Table 1) were colored black, and docked on the grid to facilitate definition of AFC in terms of location and space occupied. Examples of AFC isoforms are highlighted using boxes or circles on the gird. Identical substructures (AFC isoforms) were highlighted using boxes or circles of the same color. The effect of both *cis*- and *trans*-amino acid substitutions was reflected on the AFC isoforms observed on each virus HA1 surface. AFC isoforms observed in the grid locations studied were used for complementing structural units (CSU) calculation (Section 2.6, Table 2, Figure 4).

Several conserved substructures were selected to anchor our view in, or around, adjacent locations on the surface of the HA1 molecule. These conserved substructures were termed “anchor sites” (AS). Visualization of other discrete substructures was enhanced by coloring VS and AS, application of a grid, and unifying rotation angle (Figure 3). This visualization enhancement technique facilitated the description of the surface substructures of the HA molecule in terms of shape and space occupied (Figure 3).

Eight AS were selected for comparison purposes (AS-I to AS-VIII) (Table 1). AS-I, AS-III, and AS-V were used to dock the 3D model of HA1 on the grid (Table 1, Figure 3). Comparisons made in Case C allowed the recognition of AFC that may have led to the ability of the virus (Egypt/2014) to escape the vaccination shield. An example of these configuration changes are those found in the grid areas formed by cells (2D, 2E, 3D, and 3E), (8D, 8E, 9D, and 9E), (6A, 6B, 7A, and 7B), and (5E, 5F, 6E, and 6F); all four are parts of known VS (Figure 3). Change in the glycosylation motif formed by amino acids 154–156 and 72–74 [22,47] of the AFC defined by the first two grid areas probably added to the phenomenon of escape from vaccine shield in Case C. Acquisition of N-glycans on the globular head of the HA can mask or modify antigenic sites recognized by nAbs [48].

The functional configuration defined by cells (5E, 5F, 6E, and 6F; Figure 3) is actually formed by a conserved amino acid (H125) that changes in response to mutations in neighboring amino acids of the field virus (Egypt/2014) of Case C (Figure S1, Figure 2). This illustrates the usefulness of the described approach in visualizing the effect of both *cis*-, and *trans*-amino acid substitutions on particular substructures.

Inclusion of other field virus HA models in the comparison revealed that multiple configurations were permissible for the RBS (magenta areas from columns A–D); all modeled HA molecules came from infectious viruses (Figure 2, Table 3). This permissiveness for variation in RBS configuration supported the idea of AFC existence between viruses. Moreover, the comparison revealed the existence of AFC that have a distinctly limited number of alternative configurations within the field virus and vaccine seed collective pool, e.g., the structure pointing diagonally towards cell 1B from cell 2C in the grid areas formed by cells 1B, 1C, 2B, and 2C (Figure 3). The latter substructure is preserved in the Egyptian field viruses reported in this study.

The exhibited limitation of AFC of a specific substructure of the molecule (isoforms) within the pool of studied viruses happens despite the continued evolution of quasi-species spectrum; as a consequence of fulfilling the FC. Selection of an expressed mutation (AFC isoform) in the quasi-species pool is always under the influence of the “current” vaccine shield/population immunity. Once a change in vaccine shield/population immunity has occurred, immunity drives the selection from the allowable AFC isoforms to facilitate virus survival/escape within this immunocompetent population.

### 2.6. Visualization of Hemagglutinin (HA) AFC Facilitates Complementing Structural Units (CSU) Selection from Available Vaccines to Protect against Emerging Avian Influenza Virus (AIV) Strains

Proper comparisons between the 3D models of the HA1 of a circulating strain and available commercial vaccine seed viruses can facilitate rapid recognition of vaccines capable of exposing the immune system to the emerging virus AFC isoform pool. Moreover, since the vaccine seed AFC isoform pool constituting the totality of its HA1 surface configuration is fixed, it would then be possible to overcome the deficiency of a given vaccine in use with another that complements the sum of the AFC isoforms on the emerging virus. The different AFC isoforms of AIV HA1 present in commercial vaccines can then be considered as complementing structural units (CSU).

Previous studies have provided evidence of a synergistic action of two or more mAbs in prevention of escape mutants generation, and enhancement of the efficacy of passive therapy against H5N1 infection [33]. It was also argued that combination therapy with synergistic mAbs may allow for a lower dose of antibody to be administered for passive therapy of influenza infection [33]. Due to the clonal nature of the adaptive immune response, it is predicted that this synergistic effect will also be observed upon use of the CSU of different current commercial vaccines (i.e., a vaccine cocktail). Understandably, an optimization of the relative concentrations of each vaccine used is required to ensure a balanced exposure of immune system to the sum of AFC isoforms constituting the surface of the emerging virus. The CSU will be the focus of optimization since the majority of the antigen content is formed of conserved structural units.

**Figure 4:**
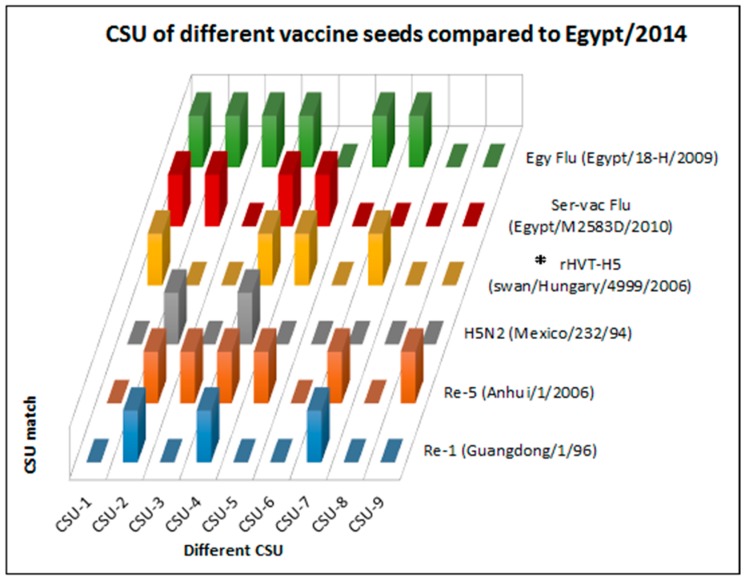
Complementing structural units (CSU) selection from vaccine seed AFC isoforms. The AFC isoform pool of Case C field virus HA1 was identified on a docked 3D model according to the method adopted for CSU definition (9 grid locations were selected to make comparisons; Table 2). Each field virus AFC isoform was examined in relation to comparable structures on the different vaccine seed viruses in this study (Section 3.1 and Section 3.2), and two vaccine seed viruses that use the HA of Egyptian field viruses (Egy Flu and Ser-Vac Flu; Figure S4). Every AFC isoform allowed was given a value (e.g., Guangdong/1/96 grid location 8B = a, Anhui/1/06 grid location 8B = b, H5N2 grid location 8B = c, rHVT/AI-H5 grid location 8B = b). In the latter example, the substructure was considered CSU-5. Simple calculations made on a Microsoft^®^ Excel^®^ worksheet (Worksheet S1) were used to compare the AFC isoforms pools on the field virus studied and on the available vaccine seed HA molecules (Section 3.6). Comparisons showed which regions have the same surface structure on both the field virus and the vaccine seed. Comparisons also showed which CSU were needed to cover the remaining field virus AFC isoform pool, and on which vaccine seed HA1 they were present. Results showed that the vaccine used in Case C (marked with an asterisk) represented a fraction of the AFC isoforms expressed on the HA1 of Egypt/2014 outbreak virus. Five CSU were needed to complete the pool of expressed AFC recognized in this study. The same analysis showed that Re-5 (A/Anhui/1/6) seed contained additional CSU (CSU-2, CSU-3, and CSU-9) that would have helped in broadening the polyclonal responses of vaccinated birds to Case C virus. Egy Flu contained additional CSU (CSU-2, CSU-3, and CSU-6) and its use would have also broadened the polyclonal responses of vaccinated birds.

Comparisons between properly aligned anchored views of the 3D models of the HA1 of circulating strains and commercial vaccine seed viruses helped define CSU on grid addresses/areas where AFC were clearly distinguished (nine grid locations were selected to make comparisons; Table 2, Figure 3). Every AFC isoform allowed was given a value (e.g., Guangdong/1/96 grid 8B = a, Anhui/1/06 grid 8B = b, H5N2 grid 8B = c, rHVT/AI-H5 grid 8B = b; CSU-5). Simple calculations made on a Microsoft Excel worksheet (Worksheet S1) were used to compare the AFC isoforms on field viruses and the CSU on vaccine seed HA1. The results were then plotted to show which vaccine combination can be used to cover the expressed AFC isoforms pool on the emerging virus targeted (Figure 4).

Application of the presented concept for the study of Case C revealed several advantages of the technology. First, the vaccine used represented only a fraction of the AFC isoforms expressed on the HA1 of Egypt/2014 outbreak virus (Table 3; Figure 4); 5 CSU were needed to complete the pool of expressed AFC recognized in this study. Second, a vaccine containing Anhui/1/06 (Re-5) seed would have improved the birds’ chances of protection against Egypt/2014 by exposing the immune system to additional CSU (CSU-2, CSU-3, and CSU-9) (Figure 4).

Understandably, vaccine seed viruses that were not included in this study thus far could provide similar, or even additional, CSU. For this reason, two vaccine seed viruses that use the HA of Egyptian field isolates (Egy Flu and Ser-Vac Flu; Figure S4) were included in the CSU analysis. Of the two, the analysis showed that Egy Flu contained additional CSU (CSU-2, CSU-3, and CSU-6) and its use would have also broadened the polyclonal responses of vaccinated birds (Figure 4).

Finally, this analysis proposes additional reasons for vaccine failure in Case C. The latter is in contradiction to the complete protection, and absence of shedding reported by the authors of Case C [54].

The presented CSU definition approach will pave the way for automation of CSU topology recognition and identification using a cube system for depth measurement and generation of CSU scores. It is envisioned that the proposed system will be based on algorithms used in face recognition, or those used in calculations of the surface accessibility view.

It is projected that expansion of the current database of vaccine HA1 3D models will increase the CSU repertoire. In addition, accumulation of 3D comparison data for HA molecules of vaccine seed viruses and virulent escape mutants will pave the ways for accurate in silico prediction of the need to update the vaccine seed, or the use of complementing vaccines in endemic areas. An emergency response would then be possible following rapid sequence analysis, modeling, and selection of complementing vaccines for simultaneous vaccination; the temporal and monetary cost of which will be significantly smaller compared to current response approaches.

Another aspect of importance is a projected reduction of the “leak” in “leaky vaccines”. A broader response to the emerging AIV AFC pool will further reduce virus shedding, and limit the chances of transmission from infected birds to other species and humans. Implementation of the presented concepts will save valuable lives by altering conditions favorable of pandemic strain generation and improving international AIV emergency response efforts.

## 3. Materials and Methods

### 3.1. Field Cases of Highly Pathogenic (HP) Avian Influenza (AI) in Vaccinated Flocks

Three H5N1 AI viruses were previously characterized following isolation from three vaccinated flocks (two layers and one broiler) in different Egyptian localities. The three flocks received different AIV vaccines (Table 3) prior to the development of AI-like disease (characterized by high mortalities, drop in production, and severe respiratory signs accompanied by external and internal hemorrhagic lesions). For comparison purposes, the terms “Case A”, “Case B”, and “Case C” were used to describe the flock, and the AIV H5N1 isolated from it (Table 3).

### 3.2. HA Sequences of Vaccine Seed and Egyptian Field Viruses Studied

Published H5N1-HA gene sequences were downloaded from GenBank (National Center for Biotechnology Information “NCBI”) [57] or EpiFlu (Global Initiative on Sharing All Influenza data “GISAID”) [58]. The vaccine seed virus sequences used in cases A and B were A/chicken/Mexico/232/94 (H5N2; GenBank accession number: AY497096; abbreviated name used: Mexico/232/94) (Table 3). In Case C, where the rHVT/AI-H5 vaccine was used, the sequence origin is A/mute swan/Hungary/4999/2006 strain [59] (H5N1; GenBank accession number: KP969039; abbreviated name used: Hungary/4999/2006) with some modifications (European patent, EP 2419132 B1 [60]).

Four reference sequences were used as representatives of AIV H5N1 Re-1, Re-5 , SER-VACC FLU, and Egy FLU vaccine seed viruses, these were A/goose/Guangdong/1/96 (GenBank number: AF144305; abbreviated name used: Guangdong/1/96), A/duck/Anhui/1/06 (GenBank number: HM172115; abbreviated name used: Anhui/1/06), A/duck/Egypt/M2583D/2010 (GenBank number: CY099580; abbreviated name used: Ser-Vac), and A/chicken/18-H/2009 (H5N1; GenBank number: CY062601; abbreviated name used: Egy Flu), respectively [5,19]. The field viruses A/chicken/Egypt/1575S/2015 (H5N1; EpiFlu number: EPI573317; abbreviated name used: Egypt/2015) and A/Egypt/N0001/2015 (human H5N1; GenBank accession number: KP864432) were also used.

### 3.3. Sequence Analysis and Phylogeny

The H5 numbering was used to describe amino acid positions of HA sequences [61,62]. A total of 1437 nucleotides from each virus, encoding its HA, were used in sequence analysis and phylogenetic tree construction. Multiple alignments and sequence identity matrix calculations were done using MegAlign™ (DNA Star, Lasergene^®^, Version 7.1.0, DNAStar Inc., Madison, WI, USA). Phylogenetic trees were calculated using the Neighbor-joining method in ClustalW [63]. Bootstrapping values were calculated using a random seeding value of 111.

In addition to the sequences described in Section 3.2, the phylogenetic analysis was done using 94 AIV H5N1 nucleotide sequences from GenBank [57] and GISAID EpiFlu [58] representing Egyptian H5N1 viruses from 2005 to 2015. BLASTN 2.2.1+ [64] was used to aid in strain selection. With the exception of replacing slashes with a dash, sequence names were used as they appear in GenBank and GISAID EpiFlu databases. Mexico/232/94 (the H5N2 vaccine seed) was not used in the phylogenetic analysis to avoid loss of branch discrimination. Allocation into clades and subclades of AIV H5N1 in the phylogeny were done according to the recent classification [65,66].

### 3.4. Two Dimensional Analysis

The antigenic indices of HA sequences were calculated using Protean™ (DNA Star, Lasergene^®^, Version 7.1.0, DNAStar Inc., Madison, WI, USA). The antigenic index was used in 2D analysis because of its relevance to vaccine design and evaluation [29].

### 3.5. Three-Dimensional Modeling

Three-dimensional model generation was done using I-TASSER [40,42] and Phyre2 [45] web portal for protein homology-based modeling, prediction, and analysis [36]. The amino acid sequence of the whole HA molecule was used for model calculations. Modeled structures were visualized using Accelrys^®^ Discovery Studio v4.1 software (Dassault Systèmes BIOVIA Corp., San Diego, CA, USA).

### 3.6. CSU Analysis

Three-dimensional models were docked on a grid after visualization angel synchronization. HA1 substructures were defined by shape within grid positions. For this analysis, nine grid locations were selected to make comparisons (Table 2). Each field virus AFC isoform was examined in relation to comparable structures on the different vaccine seed viruses examined in this study (Section 3.1 and Section 3.2), including two vaccine seed viruses that use the HA of Egyptian field viruses (Egy FLU and Ser-Vac Flu; Figure S4). Every AFC isoform allowed was given a value (e.g., Guangdong/1/96 grid location 8B = a, Anhui/1/06 grid location 8B = b, H5N2 grid location 8B = c, rHVT/AI-H5 grid location 8B = b). In the latter example, the substructure was considered CSU-5. Simple IF statements were written using Microsoft^®^ Office Home and Student 2013 Excel^®^ software so that a one would be recorded if compared substructures within a given grid location were homologous; otherwise a zero would be recorded. Tables showing the results of comparisons between studied viruses’ substructures at every studied grid location were then plotted as solid blocks (Worksheet S1).

## 4. Conclusions

Vaccine seed update is the optimal approach to providing protection against emerging AIV strains. However, the success of vaccine application is often limited by the logistics of vaccine production and delivery to affected areas. Established knowledge related to the molecular biology and evolution of AIV was utilized to propose a rapid and feasible intervention tool in the face of emerging viruses.

It was argued that only a finite number of HA1 surface substructure isoforms/AFC is possible to preserve the fitness condition within AIV subtypes. A simple approach to define AFC that are vulnerable to neutralizing antibody binding was described using 3D homology based modeling. The technique developed was also used to define homologous substructures on the HA1 of several commercial vaccine seed viruses. It was then hypothesized that CSU can be found in the HA1 AFC pool of vaccines already in use in the local market. It was further hypothesized that vaccine cocktails required to effectively expose the immune system to the emerging virus AFC isoform pool can be selected quickly following rapid sequence analysis of the emerging strain and available vaccines. CSU-based plots were used to predict which commercial vaccine combinations could have been used to cover the nine selected AFC isoforms on a recent Egyptian HP AIV H5N1 outbreak virus. In addition, it was argued that the proposed in silico approach can also improve the ability to predict the need to update vaccine seed viruses. It was projected that an improved emergency response utilizing this approach will save valuable lives by reducing the temporal and monetary costs of AIV control.

## Figures and Tables

**Table 1:** Avian influenza virus hemagglutinin subunit 1 (AIV HA1) anchor sites (AS).

Anchor Site	Amino Acid Positions	Grid Address ^1^	Reported Significance of the Region
AS-I	N220, G221, Q222	4A, 4B	Associated with replication, transmission, and receptor binding [49]
AS-II	V131	5B, 5C, 6C	Involved in receptor binding [50]
AS-III	G139	A6, 7A	Neutralizing epitope [51]
AS-IV	Y271	9B, 9C	Adjacent to amino acid 272 which affects receptor binding affinity [52]
AS-V	N72	9D	Glycosylation site [47]
AS-VI	Q115, P118	7E, 7F, 7G, 8E, 8F	Conserved epitope involved in neutralization [53]
AS-VII	T167	8H	Part of a conserved epitope involved in neutralization [53], and glycosylation [47]
AS-VIII	K161	4H	Part of a conserved epitope involved in neutralization [53]

^1^ Location on grid according to Figure 3.

**Table 2:** Spatial definition of complementing structural units (CSU) on grid.

CSU	Cell Addresses/Grid Area
CSU-1	1B, 1C, 2B, 2C
CSU-2	2D, 2E, 3D, 3E front
CSU-3	2F, 3F, 4F
CSU-4	3B
CSU-5	8B
CSU-6	8D, 8E, 9D, 9E
CSU-7	4G, 4H, 5G, 5H
CSU-8	6F, 7F over anchor site (AS)-VI
CSU-9	6A, 6B, 7A, 7B

**Table 3:** Egyptian avian influenza (AI) H5N1 viruses isolated from vaccinated flocks.

Case	A	B	C
Virus Name	A/chicken/Egypt/2010 (H5N1)	A/chicken/Egypt/13VIR3729-4/2013 (H5N1)	A/chicken/Egypt/Fadllah-7/2014 (H5N1)
Virus designation	Egypt/2010	Egypt/2013	Egypt/2014
HA gene GenBank accession number	KP869097	KF715072	KP326324
Isolation source	Layers	Broilers	Layers
Isolation date	Feb-2010	Apr-2013	Nov-2014
AIV vaccine used	Inactivated H5N2 ^1^ vaccine	Inactivated H5N2 vaccine	rHVT/AI-H5 ^2^
HI GMT before or at AI infection	9.7 Log2	High to homologous vaccine	6.4 Log2
Reference	[55]	[56]	[54]

^1^ Avian influenza virus subtype H5N2; ^2^ Recombinant herpesvirus of turkey expressing the AIV H5 vaccine seed virus (rHVT/AI-H5).

## Supplementary Materials

Supplementary materials can be found at www.mdpi.com/1422-0067/18/4/766/s1.

## Abbreviations

1D	One dimension
2D	Two dimension
3D	Three dimension
AFC	Alternative functional configurations
AI	Avian influenza
AIV	Avian influenza virus
AS	Anchor Sites
CASP	Critical Assessment of protein Structure Prediction
CDR	Complementarity determining regions
CSU	Complementing structural units
FC	Fitness condition
GISAID	Global Initiative on Sharing All Influenza data
GMT	Geometric mean titer
HA	Hemagglutinin
HI	Hemagglutination inhibition
HP	Highly pathogenic
IAV	Influenza A virus
KDa	Kilo Dalton
mAbs	Monoclonal antibodies
nAbs	Neutralizing antibodies
NCBI	National Center for Biotechnology Information
PDB	Protein Data Bank
RBS	Receptor binging site
VS	Vulnerable sites
