# Supplementary material for: Visualization of Alternative Functional Configurations of Influenza Virus Hemagglutinin Facilitates Rapid Selection of Complementing Vaccines in Emergency Situations
Source: Int J Mol Sci. 2017 Apr 4;18(4):766. doi: 10.3390/ijms18040766 (PMC5412350; doi:10.3390/ijms18040766)
Supplement: Supplementary file 1 [file ijms-18-00766-s001.zip › Figures S1-S4.pdf]

```
                                                               +---------+---------+---------+
                                                               30        40        50        60
                                                               +---------+---------+---------+
A-goose-Guangdong-1_1996                                       QDILEKTHNGKLCDLNGVKPLILRDCSVAGW    60
A-chicken-Mexico-232-94.H5N2                                   ......E...R..S.K.......K.......    60
A-chicken-Egypt-2010                                           ...............D.........G.....    60
A-chicken-Egypt-13VIR3729_4-2013                               .............N.D...............    35
rHVT-AIV_H5                                                    ...............D...............    60
A-chicken-Egypt-Fadllah_7-2014                                 ...............D...............    60
A-chicken-Egypt-1575S-2015                                     .............N.D...............    60
A-Egypt-N0001-2015                                             .............N.D...............    60
A-swan-Hungary-4999-2006                                       ...............D...............    60

                                  ---------+---------+---------+---------+---------+---------+
                                           70        80        90        100       110       120
                                  ---------+---------+---------+---------+---------+---------+
A-goose-Guangdong-1_1996          LLGNPMCDEFINVPEWSYIVEKASPANDLCYPGDFNDYEELKHLLSRTNHFEKIQIIPKS    120
A-chicken-Mexico-232-94.H5N2      ..........L...........DN...G.....N..........M.S.........F.R.    120
A-chicken-Egypt-2010              ....Q.....P..S........IN.........N.............I.R..........    120
A-chicken-Egypt-13VIR3729_4-2013  ..........L...........IN.........N.............I...........D     95
rHVT-AIV_H5                       ..........L...........IN.........N.............I............    120
A-chicken-Egypt-Fadllah_7-2014    ..........P..S........IN.........N..N..........I.R.........N    120
A-chicken-Egypt-1575S-2015        ..........L...........IN.........N.............I...........D    120
A-Egypt-N0001-2015                ..........L...........IN.........N.............I...........D    120
A-swan-Hungary-4999-2006          ..........L...........IN.........N.............I............    120

                                  ---------+---------+---------+---------+---------+---------+
                                           130       140       150       160       170       180
                                  ---------+---------+---------+---------+---------+---------+
A-goose-Guangdong-1_1996          SWSNHDASSGVSSACPYHGRSSFFRNVVWLIKKNSAYPTIKRSYNNTNQEDLLVLWGIHH    180
A-chicken-Mexico-232-94.H5N2      .................N................NV.Q....T.....I....I......    180
A-chicken-Egypt-2010              ..PD.E..L........Q.GP..Y..........NT.....E......P...........    180
A-chicken-Egypt-13VIR3729_4-2013  ...D.E..-........Q............T...D......K..................    154
rHVT-AIV_H5                       ...D.E...........Q...............DN.........................    180
A-chicken-Egypt-Fadllah_7-2014    ..PD.E..L........Q.KP..Y..........TT.....E..H...............    180
A-chicken-Egypt-1575S-2015        ...D.E..-........Q............T...D......K..................    179
A-Egypt-N0001-2015                ...D.E..-........Q............T...D......K..................    179
A-swan-Hungary-4999-2006          ...D.E...........Q...............DN.........................    180

                                  ---------+---------+---------+---------+---------+---------+
                                           190       200       210       220       230       240
                                  ---------+---------+---------+---------+---------+---------+
A-goose-Guangdong-1_1996          PNDAAEQTKLYQNPTTYISVGTSTLNQRLVPEIATRPKVNGQSGRMEFFWTILKPNDAIN    240
A-chicken-Mexico-232-94.H5N2      .......I......N..V..........SI.......................R...S.T    240
A-chicken-Egypt-2010              ..NE....R..K.SN................K....S........V........S.....    240
A-chicken-Egypt-13VIR3729_4-2013  ........R......................K....S.................S.....    214
rHVT-AIV_H5                       ........R......................K....S.......................    240
A-chicken-Egypt-Fadllah_7-2014    ...D....RI.K...................K....S........V........S..T..    240
A-chicken-Egypt-1575S-2015        ........R......................K....S.................S.....    239
A-Egypt-N0001-2015                ........R......................K....S.................S.....    239
A-swan-Hungary-4999-2006          ........R......................K....S.......................    240

                                  ---------+---------+---------+---------+---------+---------+
                                           250       260       270       280       290       300
                                  ---------+---------+---------+---------+---------+---------+
A-goose-Guangdong-1_1996          FESNGNFIAPEYAYKIVKKGDSAIMKSELEYGNCNTKCQTPMGAINSSMPFHNIHPLTIG    300
A-chicken-Mexico-232-94.H5N2      ...T............I............N....DA.....V...........V..F...    300
A-chicken-Egypt-2010              ...........N..........T......K.S.........I..................    300
A-chicken-Egypt-13VIR3729_4-2013  ...........N..........T........S.........I..................    274
rHVT-AIV_H5                       ...........N..........T....K.............I..................    300
A-chicken-Egypt-Fadllah_7-2014    ...........N..........T...........S......V..................    300
A-chicken-Egypt-1575S-2015        ...........N..........T........S.........I..................    299
A-Egypt-N0001-2015                ...........N..........T........S.........I..................    299
A-swan-Hungary-4999-2006          ...........N..........T....K.............I..................    300
```

Figure S1. Multiple alignment of AIV HA1 amino acid sequences. Field and vaccine seed H5 isolates were aligned to show amino acid substitution locations. Multiple alignments and sequence identity matrix calculations were done using MegAlign™ (DNA Star, Lasergene®, Version 7.1.0, DNAStar Inc., Madison, WI, USA). The H5 numbering system was used. Sequence elements involved in VS formation are colored green, magenta, blue, and red, according to the original coloring code of VS1-4, respectively. The brown box refers to a linear epitope. Dots indicate amino acid similarity.

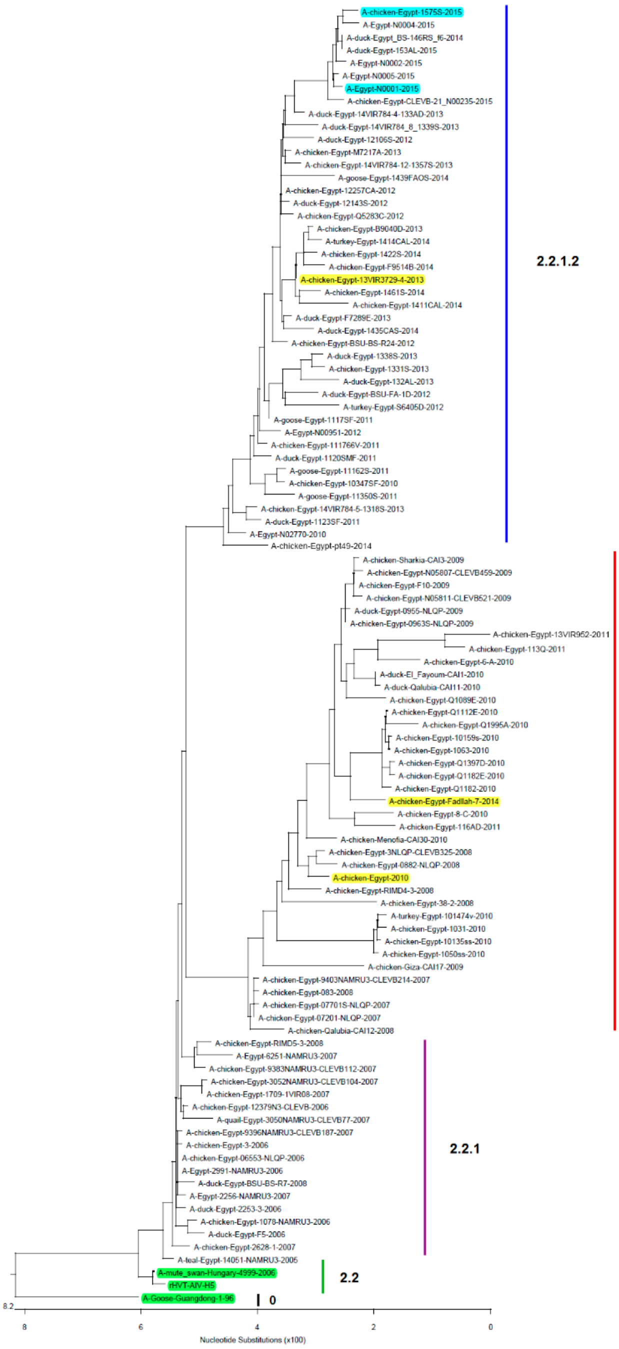

Figure S2. Phylogenetic relationships between AIV H5N1 HA of field and vaccine seed viruses. Phylogeny was conducted to investigate whether the outbreaks in the studied flocks were due to either the emergence of vaccine escape mutants from endemic AIV strains, or the introduction of heterologous AIV. The nucleotide sequences of 1430 base fragments of the AIV HA were used to generate the trees. Multiple alignments and sequence identity matrix calculations were done using MegAlign™ (DNA Star, Lasergene®, Version 7.1.0, DNAStar Inc., Madison, WI, USA). Phylogenetic trees were calculated using the Neighbor-joining method in ClustalW. Bootstrapping values were calculated using a random seeding value of 111.The phylogenetic analysis was done using 94 sequences in addition to the field and vaccine seed viruses described in Section 3.2. Viruses represented the Egyptian AIV H5N1 from 2005 to 2015. Mexico/232/94 (the H5N2 vaccine seed) was not used in the phylogenetic analysis to avoid the loss of branch discrimination. Case A, B, and C viruses that branched as previously reported indicate proper phylogeny. In two cases, A and B, the outbreak viruses belonged to clades 2.2.1.1 and 2.2.1.2, respectively. Hungary/4999/2006, the rHVT/AI-H5 vaccine seed virus, was more closely related to the case C field virus.

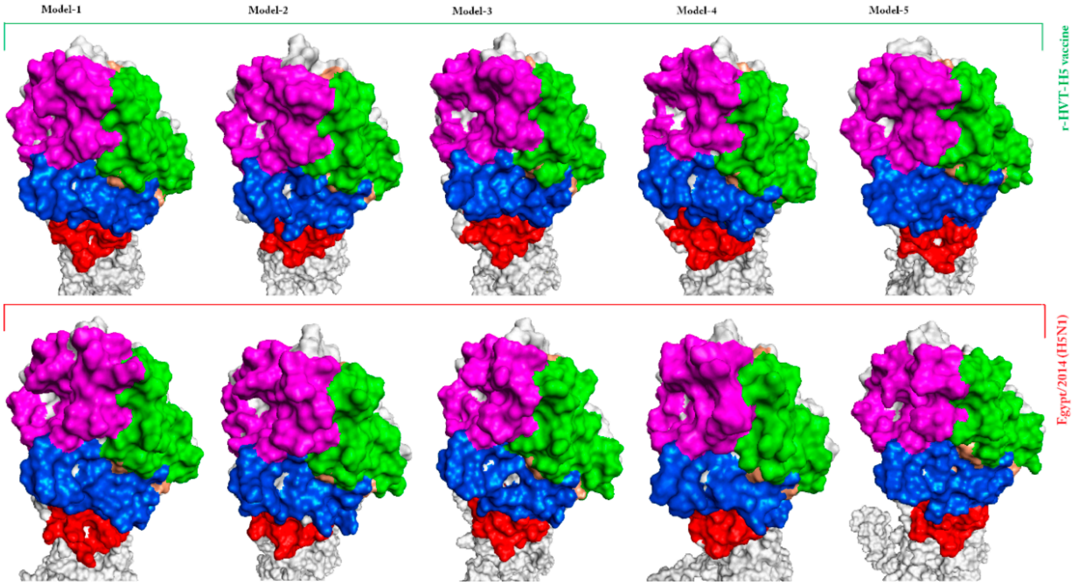

Figure S3. I-Tasser-predicted 3D structure variations. I-Tasser was used to generate 3D models for the HA molecules of Egypt/2014 and rHVT/AIV-H5 vaccine seeds. Five alternative 3D models were generated for each. Modeled structures were visualized using Accelrys® Discovery Studio v4.1 software (Dassault Systèmes BIOVIA Corp., San Diego, CA, USA). (Section 3.5). Sequence elements involved in VS formation were colored green, magenta, blue, and red, according to the original coloring code of VS1-4, respectively. The linear epitope sequences were colored bronze. Models were synchronized and trimmed to present an optimum view of the VS. The alternative 3D models generated using I-TASSER 3D had discernible substructures, albeit not in a manner that facilitates comparisons between different molecules. These multiple alternative folding patterns and interactions with solvent were observed on both the HA1 and the stalk regions of the modeled molecules.

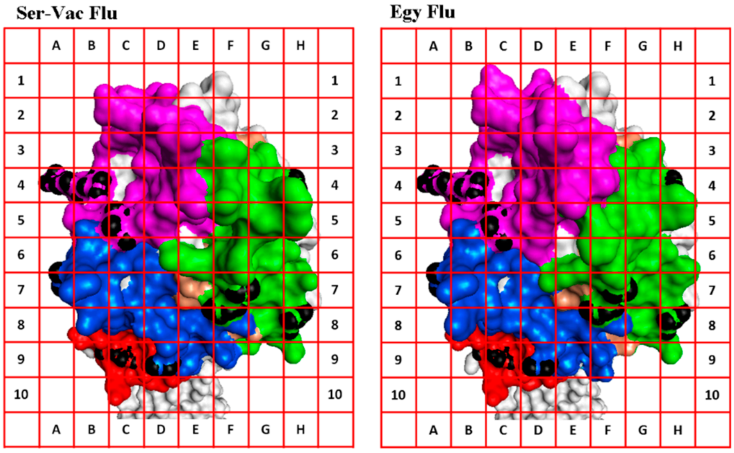

Figure S4. Visualization of AIV HA1 AFC of vaccine seed viruses expressing the HA of Egyptian field viruses. Three-dimensional models of whole HA molecules of seed viruses used in Egy Flu and Ser-Vac Flu were generated using Phyre2. The whole HA sequence was used for modeling. Modeled structures were visualized using Accelrys® Discovery Studio v4.1 software (Dassault Systèmes BIOVIA Corp., San Diego, CA, USA). (Section 3.5). Sequence elements involved in VS formation were colored green, magenta, blue, and red, according to the original coloring code of VS1-4, respectively. The linear epitope sequences were colored bronze. Models were synchronized and trimmed to present an optimum view of the VS. Conserved substructures identified as AS (Table 1) were labeled black, and docked on the grid to facilitate definition of AFC in terms of location and space occupied. Nine grid addresses/areas with clearly defined AFC were used for comparisons and CSU definition (Table 2).
