# Supplementary material for: Visualization of Alternative Functional Configurations of Influenza Virus Hemagglutinin Facilitates Rapid Selection of Complementing Vaccines in Emergency Situations
Source: Int J Mol Sci. 2017 Apr 4;18(4):766. doi: 10.3390/ijms18040766 (PMC5412350; doi:10.3390/ijms18040766)
Supplement: Supplementary file 1 [file ijms-18-00766-s001.zip › Table S1.pdf]

Table S1. Amino acid variations in the vulnerable sites and linear epitope regions of the HA of three Egyptian HPAI viruses isolated from vaccinated flocks compared to the vaccine seed viruses used in vaccination.

HA region (aa number*)		Egypt/2010[C]	Egypt/2013[C]	Mexico/232/94	Egypt/2014[D]	rHVT/AI-H5
	(117)	I hb, =	I hb, =	F hb, =	I hb, =	I hb, =
	(119)	K hl, +	K hl, +	R hl, +	K hl, +	K hl, +
	(120)	S hl, =	***D hl, -***	S hl, =	N hl, =	S hl, =
	(123)	***P hb, =***	S hl, =	S hl, =	***P hb, =***	S hl, =
	(124)	***D hl, -***	***D hl, -***	N hl, =	D hl, -	D hl, -
VS-1[A]	(158)	***P hb, =***	***P hb, =***	Q hl, =	P hb, =	P hb, =
	(162)	***E hl, -***	K hl, +	R hl, +	***E hl, -***	R hl, +
	(163)	S hl, =	S hl, =	T hl, =	S hl, =	S hl, =
	(165)	N hl, =	N hl, =	N hl, =	***H hl, +***	N hl, =
	(169)	P hb, =	***Q hl, =***	I hb, =	Q hl, =	Q hl, =
	(126)	E hl, -	E hl, -	D hl, -	E hl, -	E hl, -
	(129)	***L hb, =***	***Deletion***	S hl, =	***L hb, =***	S hl, =
	(151)	I hb, =	***T hl, =***	I hb, =	I hb, =	I hb, =
	(154)	N hl, =	N hl, =	N hl, =	***N hl, =***	D hl, -
	(155)	N hl, =	***D hl, -***	N hl, =	T hl, =	N hl, =
VS-2[A]	(156)	***T hl, =***	A hb, =	V hb, =	***T hl, =***	A hb, =
	(183)	***N hl, =***	D hl, -	D hl, -	D hl, -	D hl, -
	(184)	***E hl, -***	A hb, =	A hb, =	***D hl, -***	A hb, =
	(188)	***T hl, =***	***T hl, =***	I hb, =	T hl, =	T hl, =
	(189)	R hl, +	R hl, +	K hl, +	R hl, +	R hl, +
	(190)	L hb, =	L hb, =	L hb, =	I hb, =	L hb, =
	(71)	P hb, =	L hb, =	L hb, =	P hb, =	L hb, =
	(74)	***S hl, =***	P hb, =	P hb, =	***S hl, =***	P hb, =
	(110)	R hl, +	H hl, +	H hl, +	R hl, +	H hl, +
VS-3[A]	(138)	Q hl, =	Q hl, =	N hl, =	Q hl, =	Q hl, =
	(140)	***G hb, =***	R hl, +	R hl, +	K hl, +	R hl, +
	(141)	***P hb, =***	S hl, =	S hl, =	***P hb, =***	S hl, =
	(144)	***Y hl, +***	F hb, =	F hb, =	***Y hl, +***	F hb, =
	(43)	***D hl, -***	N hl, =	S hl, =	D hl, -	D hl, -
VS-4[A]	(45)	***D hl, -***	***D hl, -***	K hl, +	D hl, -	D hl, -
	(53)	R hl, +	R hl, +	K hl, +	R hl, +	R hl, +
	(270)	***K hl, +***	***E hl, -***	N hl, =	E hl, -	E hl, -

	(272)	***S***$^{hl, =}$	***S***$^{hl, =}$	G$^{hb, =}$	G$^{hb, =}$	G$^{hb, =}$
	(275)	***N***$^{hl, =}$	***N***$^{hl, =}$	D$^{hl, -}$	***S***$^{hl, =}$	N$^{hl, =}$
	(276)	***T***$^{hl, =}$	***T***$^{hl, =}$	A$^{hb, =}$	T$^{hl, =}$	T$^{hl, =}$
Linear[B]	(244)	N$^{hl, =}$	N$^{hl, =}$	T$^{hl, =}$	N$^{hl, =}$	N$^{hl, =}$
	(252)	***N***$^{hl, =}$	***N***$^{hl, =}$	Y$^{hl, +}$	N$^{hl, =}$	N$^{hl, =}$

[A] Vulnerable sites as described by Zuo et al. [1]. [B] Linear epitope described by Tan et al. [2]. * The H5 numbering system was used. Amino acids involved in VS formation were colored green, magenta, blue, and red, according to the original coloring code of VS1-4, respectively [1]. Amino acid variations within the linear epitope were colored bronze. [C] Compared to Mexico/232/94 H5N2 vaccine HA. [D] Compared to rHVT-AI-H5 vaccine HA. Physical properties of the aa [3–5] were indicated as a superscript to the right of the aa designation as follows; ^{hl}: hydrophilic, ^{hb}: hydrophobic, $^{=}$: neutral, $^{+}$: positively-charged (basic), and $^{-}$: negatively-charged (acidic). Amino acids in grey shadows are different from those in the same location on the HA of the vaccine seed virus used in the vaccination of the flock from which they were isolated (Table 3). Amino acid with physical properties different than those in the same location on the vaccine seed HA are bolded and italicized.

References

1. Zuo, T.; Sun, J.; Wang, G.; Jiang, L.; Zuo, Y.; Li, D.; Shi, X.; Liu, X.; Fan, S.; Ren, H.; et al. Comprehensive analysis of antibody recognition in convalescent humans from highly pathogenic avian influenza H5N1 infection. *Nat. Commun*. **2015**, *6*, 8855.
2. Tan, Y.; Ng, Q.; Jia, Q.; Kwang, J.; He, F. A novel humanized antibody neutralizes H5N1 influenza virus via two different mechanisms. *J. Virol.* **2015**, *89*, 3712–3722.
3. Khan Academy. Available online: https://www.khanacademy.org/science/chemistry
4. Basics of Protein Structure. Available online: http://www.proteinstructures.com/Structure/Structure/amino-acids.html
5. Davidson College Website. Available online: http://www.bio.davidson.edu/courses/genomics/jmol/aatable.html
